# Medical Muss at Louisville

**Published:** 1877-01-20

**Authors:** 


MEDICAL MUSS AT LOUISVILLE.— One of the students of Dr. Gaillard's Medical College (or Colleges ?) has sued the faculty for the fees paid by him. We have condemned this "two in one" institution, as all honest men must, but we sympathize with any of our professional brethren when in trouble. We do not go with those—if any there be—who desire the annihilation of Dr. G., or the college in which he teaches. If he has done wrong, he should, and doubtless will, give ample reparation, both to the profession and the parties aggrieved. Charity becomes us, and we should be slow to condemn, utterly and entirely, until all the facts are known. We should deal justly—condemn where blame is proven, and withhold it until evil is made plain. We sincerely trust Dr. G. may clear his professional garments of all taint, even at the expense of acknowledgement of error committed. He will lose nothing by such a course, but, on the other hand, will gain largely by it.

To all engaged in this controversy, we say: Seek the injury of no man. Give justice and truth the amplest play. Bring error to the plumb-line and exact no more. Do right, but temper right with the soft smiles of charity—the sweetest, holiest principle graciously given to our fallen race. G..

